# A congenital case of nasal lipomeningocele with cerebrospinal fluid rhinorrhea

**DOI:** 10.11604/pamj.2025.52.173.49122

**Published:** 2025-12-19

**Authors:** Ankita Vinod Selkar, Bibin Kurian

**Affiliations:** 1 Child Health Nursing Department, Smt. Radhikabai Meghe Memorial College of Nursing, Sawangi (Meghe), Wardha, Maharashtra, India

**Keywords:** Nasal lipomeningocele, cerebrospinal fluid rhinorrhoea, neural tube defect, frontoethmoidal encephalocele

## Image in medicine

An 8.5-month-old male presented with a progressively enlarging midline nasal swelling since birth. The soft, non-tender, pedunculated mass extended over the upper lip and nose and was associated with intermittent clear nasal discharge suggestive of cerebrospinal fluid (CSF) rhinorrhoea. There was no history of trauma, fever, seizures, or developmental delay. The child was born full-term via vaginal delivery (3.1 kg) with an uneventful antenatal and perinatal history. No family history of congenital anomalies or consanguinity was noted. Clinical examination revealed a well-defined, transilluminant, non-pulsatile nasal mass with intermittent CSF leak. There were no other dysmorphic features or signs of raised intracranial pressure. Magnetic Resonance Imaging (MRI) of the brain and face confirmed a nasal lipomeningocele, a rare frontoethmoidal meningoencephalocele variant characterized by herniation of meninges and fatty tissue through an anterior skull base defect. This congenital neural tube defect results from failure of anterior neuropore closure during embryogenesis. The presence of CSF rhinorrhoea indicated a direct communication between the intracranial space and nasal cavity, posing a high risk of ascending infections, including meningitis. Empirical antibiotics were initiated to prevent life-threatening complications. A multidisciplinary team involving neurosurgery and plastic surgery planned surgical intervention, including excision of the lipomeningocele, repair of the skull base defect, and watertight dural closure. Early diagnosis and prompt surgical management are crucial in preventing neurological complications and ensuring favorable outcomes in such rare congenital anomalies.

**Figure 1:**
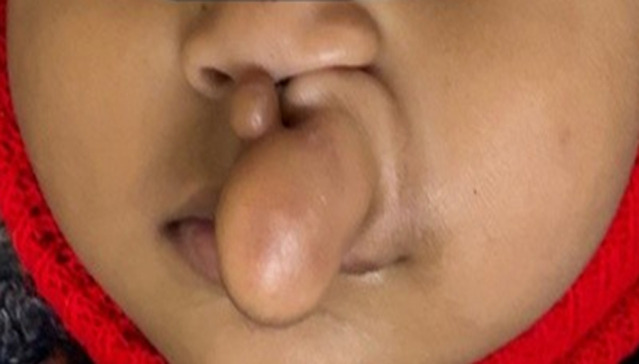
nasal lipomeningocele with cerebrospinal fluid rhinorrhoea

